# On the TRAIL of Better Therapies: Understanding TNFRSF Structure-Function

**DOI:** 10.3390/cells9030764

**Published:** 2020-03-20

**Authors:** Éva S. Vanamee, Denise L. Faustman

**Affiliations:** Immunobiology Laboratories, Massachusetts General Hospital, 13th Street, Building 149, Rm. 3602, Boston, MA 02129, USA; eva.vanamee@gmail.com

**Keywords:** TRAIL, TRAIL receptors, apoptosis, TNFSF signaling, receptor clustering, antiparallel dimer, hexagonal lattice, cancer

## Abstract

Tumor necrosis factor (TNF) superfamily ligands show diverse biological functions, such as the induction of apoptotic cell death or cell survival and proliferation, making them excellent therapeutic targets for cancer and autoimmunity. We review the latest literature on TNF receptor superfamily signaling with a focus on structure-function. Using combinatorics, we argue that receptors that cluster on the cell surface and are activated by membrane-bound ligands need to arrange in a highly ordered manner, as the probability of random ligand and receptor arrangements matching up for receptor activation is very low. A growing body of evidence indicates that antiparallel receptor dimers that sequester the ligand binding site cluster on the cell surface, forming a hexagonal lattice. Upon ligand binding, this arrangement puts the activated receptors at the right distance to accommodate the downstream signaling partners. The data also suggest that the same geometry is utilized regardless of receptor type. The unified model provides important clues about TNF receptor signaling and should aid the design of better therapies for cancer and various immune mediated diseases.

## 1. Introduction

The TNF-related apoptosis-inducing ligand (TRAIL/Apo2L) [1] is a member of the TNF superfamily (TNFSF). Members of the TNFSF signal through their corresponding family of TNF receptors (TNFRSF) (Table 1). TRAIL has generated a lot of interest as a potential cancer therapy on the basis of the observation that it can selectively induce cancer cell death. In addition, because TRAIL provides an external trigger for apoptosis, it has the potential to overcome resistance to internal triggers of apoptosis after radiation or chemotherapy. There have been many excellent reviews on TRAIL biology and the mechanism of action with implication for therapeutic applications in recent years [2,3,4,5,6,7,8]. Here, we focus on the structure-function of TRAIL and extend our discussion to other members of the TNFSF/TNFRSF to illustrate the mechanism of signaling by reviewing the most up-to-date and relevant information from the scientific literature.

## 2. Receptors and Ligands Need to Cluster in a Highly Ordered Manner on the Cell Surface for Efficient Signaling

TRAIL and other TNFSF ligands are type II membrane proteins that share the classic TNF homology domain (THD) and form noncovalent trimers [9,10]. Trimerization is essential for receptor activation and the membrane-bound ligands have been shown to signal more efficiently via their corresponding receptors compared to the cleaved, soluble ligands. The TNFRSF receptors are type I transmembrane proteins and their ectodomains contain between 1 and 6 pseudorepeats of cysteine-rich domains (CRDs) (Table 1) [10,11,12].

Experimental data support receptor oligomerization of TRAIL and other TNFRSF receptors on the cell surface but there have been conflicting models proposed over the years [4,6,13,14]. Activating several receptors all at once by their corresponding membrane-bound ligands necessitates that both the receptors and the ligands are arranged with the same geometry, which rules out random receptor/ligand arrangement on the cell surface. To illustrate this, let us assume that there are 10 separate trimeric receptors that are pre-arranged on the surface with the potential sites they can occupy represented by lattice points in a 10 by 10 matrix. We consider each trimer as a single unit. The first receptor trimer can be placed onto one of 100 potential sites of the 10 × 10 matrix. Once that place is taken, the second receptor trimer has one less, i.e., 99 potential places to occupy, and so on, resulting in 100 × 99 × 98 × 97 × 96 × 95 × 94 × 93 × 92 × 91 potential ways the 10 receptor trimers can be arranged, which can also be written as 100 factorial divided by 90 factorial (100!/90!). Because the order in which the 10 receptor trimers are placed is not important, we need to divide the above number by the permutation of 10 or 10 factorial (10!). This leads to a potential combination of the receptor arrangement referred in combinatorics as “hundred choose ten” or C (100,10) and can be calculated as 100!/(90! × 10!). Even with such a small number of receptors, there are more than 17 trillion ways to arrange them in our example. Not all are unique though, and we need to subtract cases where a simple rotation or translation can move one pattern onto another.

**Figure 1:**
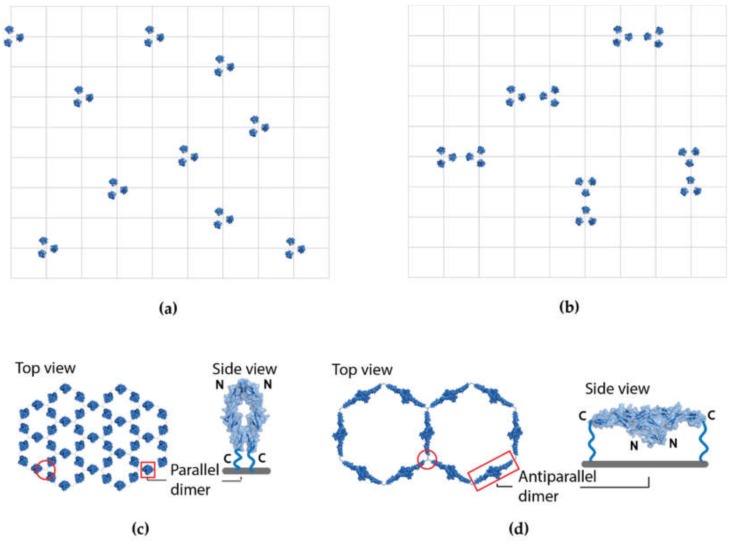
Representative arrangements of receptors on the cell surface. (**a**) An example of a random arrangement of 10 receptor trimers limited to lattice points of a 10 × 10 matrix, top view. Even with this constraint there are more than 17 trillion possible combinations (C(100,10)), more than 4 trillion of them unique. To activate these receptors with their corresponding membrane-bound ligands, the ligands would have to be arranged exactly the same way to simultaneously activate the receptors. The probability of that is less than 1 in 4 trillion. (**b**) Random arrangement of pairs of receptor trimers. Having 10 receptors arranged in pairs of trimers on the cell surface lowers the number of unique receptor arrangements to over 38 million and the probability that one of these arrangements will match up with corresponding random ligands is still very low. (**c**) Tight hexagonal lattice with parallel dimer arrangements of TNF receptor 1 (TNFR1) [Protein Data Bank (PDB) ID:1NCF] top view, and side view of a single dimer. The N and C terminal are labeled. (**d**) Hexagonal lattice with antiparallel receptor dimers of TNFR1 (PDB ID: 1NCF) top view, and side view of a single antiparallel dimer. Five times as many receptors can be fit into the same area in the tight hexagonal cluster in (**c**) compared to the larger hexagonal lattice occupied by the antiparallel receptor dimers. The red circles in (**c**) and (**d**) illustrate the trimerization interface. The molecular representations were created by the molecular graphics program PyMOL [17].

We can simplify the translation problem by subtracting patterns from the 17 trillion total that only occupy one half of the 10 × 10 matrix. Every such pattern in one half of the matrix can be translated onto a matching pattern in the other half. This can be calculated as C(50,10) = 50!/(40! × 10!) ~10 billion, a small rounding error compared to the 17 trillion total. There are other patterns that are counted more than twice, but their numbers are even smaller and can be neglected. The ~17 trillion combinations have to be divided by 4 to take into account the four 90 degree rotations that transform one pattern into another. After taking rotational and translational symmetry into account, we end up with more than 4 trillion unique patterns or unique ways the receptor trimers can be arranged. Figure 1a illustrates one such pattern. Because the membrane-bound ligands need to be arranged the exact same way as the receptors in order for them to align for binding and activation, the probability of a particular random ligand arrangement matching up with a random receptor arrangement in this example is less than 1 in 4 trillion. This illustrates the vast number of combinations even for such a small number of receptors. In reality, the number of receptors are vastly higher on the cell surface and vary depending on the type of receptor and the cell. As an example, TNF receptors can vary from a little over a hundred on T cells in healthy subjects [15] to several thousand on human neutrophils [16]. Therefore, the probability of random receptors matching up with randomly arranged membrane-bound ligands is extremely low and highly unlikely. Although it may not seem obvious, this also rules out pairs of receptor trimers randomly arranged on the cell surface (illustrated in Figure 1b) and argues for both the receptors and ligands being arranged in a highly ordered, non-random cluster with matching geometry. It is not necessary that all receptors are in one large cluster, they could be expressed in smaller groups, but all the smaller clusters will have to be highly ordered. The implications of this go beyond the TNF receptor superfamily and we believe will also apply to any receptor that clusters on the cell surface and is activated by membrane bound ligands.

Ligand-free TNFRSF receptors have been shown to form two different dimers [18,19]. One form is a parallel dimer in which the receptors are in a head-to-head arrangement, meaning that the two N-terminals line up next to each other on one end and the C-terminals on the other. This dimer form exposes the ligand binding site on the outside (Figure 1c). The other dimer form is an antiparallel or head-to-tail arrangement, in which the two receptors are connected by their N-terminal ends to each other but run in the opposite direction, separating the C-terminal ends by more than 100 Å (Figure 1d). This dimer interface buries part of the ligand binding site. It has also been shown that TRAIL and other TNFRSF receptors cluster on the cell surface and interact via their pre-ligand assembly domains (PLADs) formed by the N-terminal, CRD1 and part of the CRD2 domains [20,21,22]. Both the parallel and antiparallel dimers can multimerize on the cell surface as dimers of trimers forming hexagonal lattices [14,18]. Both hexagonal arrangements anchor the receptors as trimers in the membrane and satisfy the requirement for PLAD interactions. The parallel dimers can form a tight hexagonal cluster with no separation between receptor trimers and the antiparallel dimers can arrange in a larger hexagonal lattice where each receptor trimers separated by more than 100 Å (Figure 1c,d). As we have argued earlier, the larger hexagonal lattice is able to accommodate the downstream signaling partners with the same geometry regardless of receptor type leading to the hypotheses of a uniform signaling mechanism [14]. Next, we are going to review additional data supporting this model.

## 3. TRAIL and Its Receptors

**Figure 2:**
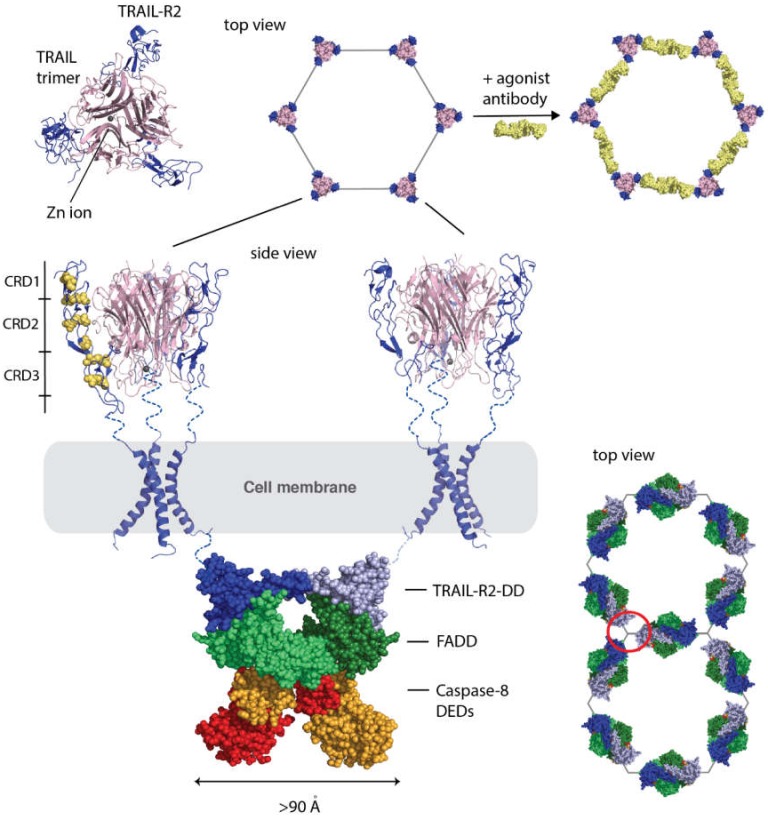
TRAIL-R2 activation and formation of the death inducing signaling complex (DISC). The TRAIL-TRAIL-R2 complex structure (PDB ID: 1DU3) is shown in cartoon representation (top left). The TRAIL ligand trimer (pink) surrounded by three TRAIL-R2 receptors (blue). These complexes can cluster on the cell surface in a hexagonal lattice (top middle) that are stabilized by agonist antibodies shown in yellow (top right). Below two TRAIL-TRAIl-R2 complexes are illustrated in side view. The three cysteine-rich domains (CRD1-3) of TRAIL-R2 are labelled in the left complex. The transmembrane domain of TRAIL-R2 is also shown (PDB ID: 6NHY). A model of the DISC complex is illustrated below the cell membrane in spheres representation. The partial model of the TRAIL death domain (TRAIL-DD) dimer is shown in dark and light blue, the Fas associated domain (FADD) dimer is shown in dark and lighter green, and the Caspase 8 death effector domain (DED) dimer is shown in red and orange. On the right a model of how the DISC may arrange in a hexagonal lattice is illustrated in top view.

Among the TNFSF, TRAIL signaling biology is one of the most complex. In addition to binding to its two death receptors, TRAIL receptor 1 (TRAIL-R1, also referred to as death receptor 4 or DR4) and TRAIL receptor 2 (TRAIL-R2/DR5), it also binds to three decoy receptors, TRAIL-R3 (also known as decoy receptor 1, DcR1) and TRAIL-R4 (also known as decoy receptor 2, DcR2) and soluble receptor osteoprotegerin (OPG) that can sequester TRAIL and therefore also functions as a decoy receptor [23,24,25,26]. The differential expression of the DcRs and TRAIL death receptors on different cell types can modulate the outcome of TRAIL function resulting in cell death of certain cell types but not of others. Similar to other TNFSF ligands, a trimeric TRAIL ligand binds to three receptor monomers to form the active signaling complex shown in Figure 2. Unique among TNFSF ligands is that TRAIL contains a Zn ion that is coordinated by a Cys residue (Cys^230^) from each monomer. The loss of Zn ion can lead to instability and loss of activity of human recombinant TRAIL (hrTRAIL) [27,28]. Several successful approaches have been utilized to overcome these challenges and to improve the cancer targeting potential of hrTRAIL that have been reviewed extensively elsewhere [3,29].

## 4. TRAIL Receptor Downstream Signaling: DISC Assembly and Caspase Interactions

TRAIL-R1/R2, similar to Fas induce apoptosis via formation of the death inducing signaling complex (DISC) that, in addition to the death domain (DD) of TRAIL contains Fas associated death domain (FADD) and the cysteine protease, Caspase-8. The Fas-DD-FADD complex structure provided insight into how these intracellular DISC components may form a hexagonal lattice after Fas ligand binding and Fas receptor activation [30]. Shen et al. more recently have reported the structure of Caspase-8 DEDs in an open and closed conformation [31]. Both structures show a dimer with a novel domain-swapped conformation in which the C-terminal helices are exchanged between the two subunits. The open conformation exposes a hydrophobic patch that may facilitate the interaction with FADD in the DISC. On the basis of these structures, we have created our model of the DISC complex. First, we modeled the TRAIl-R2-DD monomer based on the Fas-DD open conformation structure [30] using the Phyre2 server [32]. The server returned a partial structure with a 99.9% confidence interval. The TRAIL-R2 DD-FADD complex was then modeled on the basis of the Fas-DD-FADD complex [30]. We used the full FADD structure [33] to complete the TRAIL-R2-DD-FADD complex. Finally, we docked the structure of the caspase-8 DED domain-swapped open dimer structure [31]. The catalytic domains of caspase-8 were not included. The resulting model is shown in Figure 2. This is only a crude model and will likely be improved over the coming years as more experimental data become available. However, it clearly demonstrates that in order to accommodate components of the DISC, the TRAIL receptors have to be separated by ~100 Å or more. This model is further supported by data of a TRAIL-R2/DR5 agonist antibody that binds on the outside of the receptor and bridges two receptor trimers together [13] that are similarly separated by ~100 Å or more. This antibody stabilizes the receptors in the hexagonal lattice (Figure 2) and shows synergistic activity when co-administered with hrTRAIL [13,34]. This strategy in general could be employed to other TNFRSF members. Although there are no structures available of the ligand-free TRAIL receptors, the structure of TRAIL-R2 in complex with human cytomegalovirus (HCMV) UL141 shows a heterotetramer in which TRAIL-R2 is arranged in an antiparallel orientation bridged by an HCMV UL141 dimer [35]. It was proposed that HCMV UL141 blocks the transport of TRAIL-R2 from the endoplasmic reticulum (ER) to the cell surface, thus downregulating TRAIL-R2 surface expression and function. The structure indicates that the membrane proximal ends of the full TRAIL-R2 ectodomain would be separated by more than 90 Å. This indicates that TRAIL-R2 may exist as an antiparallel dimer at least in the ER. The structure of the transmembrane domain (TMD) of TRAIL-R2/DR5 is a notable exemption to the above examples, showing that the TMD can dimerize and may form tight hexagonal clusters [36]. However, this structure was solved without the ectodomain of the receptor. The ectodomain is wider by about 25 Å than the TMD and therefore would interfere with TMD hexamer formation in the full receptor. In addition, the tight cluster could not accommodate the DISC complex. The structure, if physiologically relevant, may therefore likely represent a state after receptor ectodomain shedding.

In summary, the experimental data on both the cell surface receptors and in the cytosol on DISC assembly are consistent with the hexagonal lattice model with a separation of ~100 Å or more between receptor trimers proposed earlier [13,14,30,31] but not with the tight hexagonal cluster with no separation between receptor trimers because that would leave no room for the downstream binding partners that make up the DISC. One might argue that pairs of receptor trimers could easily form tight hexamers and then be separated upon activation by the ligand or by an antibody and move further apart as necessary to accommodate the downstream signaling partners. However, as we discussed earlier, even a few pairs of receptor trimers arranged randomly would have tens of millions of ways to arrange and an extremely low probability to match up with randomly arranged membrane-bound ligands. When we speak of pairs of trimers as the minimal unit of signaling it has to be always considered in the context of a cluster of receptors with a well-defined geometry. If one starts with a tight cluster, shown in Figure 1c, the movement of a receptor trimer would inevitably affect other receptors around it. The tight cluster would have to go through a huge reorganization, moving around many receptors in the membrane after activation to accommodate the downstream binding partners resulting in loss of signaling efficiency. The most effective receptor quiescent state is one that anchors the receptors in place in the membrane with the same geometry as the downstream signaling partners requiring only conformational changes upon activation. The hexagonal model of trimers of antiparallel dimers satisfies this requirement.

## 5. TNFRSF Antiparallel Dimer Stabilization by Antibodies Effectively Block Ligand Binding

Historically, it has been challenging to design powerful antagonists against TNFRSF members. TNF receptor 2 (TNFR2) is a master switch of cell survival and proliferation [37] and, as reported earlier, antagonist antibodies targeting TNF receptor 2 (TNFR2, TNFRSF1b) can successfully downregulate immunosuppressive T regulatory cells and eliminate tumor cells that overexpress the TNFR2 oncogene for survival [38,39]. These antibodies have shown promise in several cancer models including ovarian cancer [38] and advanced Sézary syndrome, an aggressive form of cutaneous T-cell lymphoma [40]. When examining their mechanism of action, the best antagonists have been found to bind to the CRD3-CRD4 region of TNFR2 and to require either the full antibody or F(ab’)_2_ to effectively block TNF binding [38]. In addition, these antibodies do not rely on FcγR cross-linking that often weakens antagonism and, in some cases, turns antagonist antibodies into outright agonists. The data supports a mechanism where the best antagonist binds to the antiparallel dimer form of the receptor locking in the quiescent state [14,38]. In another example, an antagonist antibody to CD40 (TNFRSF5) has also been shown to bind to the antiparallel dimer form of the receptor [41]. The antibody bound as a single Fab domain making interactions to both CD40 monomers in the dimer. It was further found that the antiparallel dimer stabilization was essential for antagonism as demonstrated by a closely related antibody that only interacted with a single CD40 monomer and had agonistic activity [41]. As we discuss next, the structure of the herpes virus entry mediator (HVEM) in complex with B and T lymphocyte attenuator (BTLA) represents another example of an antiparallel arrangement with BTLA assisting HVEM oligomerization, and playing a regulatory role in HVEM function.

## 6. Regulating Receptor Function via Antiparallel Complex Formation

HVEM (TNFRSF14) functions as a molecular switch modulating B and T lymphocyte activation [42], dendritic cell proliferation [43] and protecting mucosal epithelia from damage during inflammation [44]. In the cytosol it interacts with several TNF receptor associated factor (TRAF) homologs including TRAF2 to induce NFκB activation. LIGHT and LT- α are the two canonical TNFSF ligands that activate HVEM. BTLA and CD160 are the non-conventional ligands that modulate HVEM function. In addition, HVEM also interacts with viral proteins. Indeed, HVEM was first identified as a receptor that facilitates the entry of herpes simplex virus (HSV) via its interaction with HSV glycoprotein D (gD) [45].

**Figure 3:**
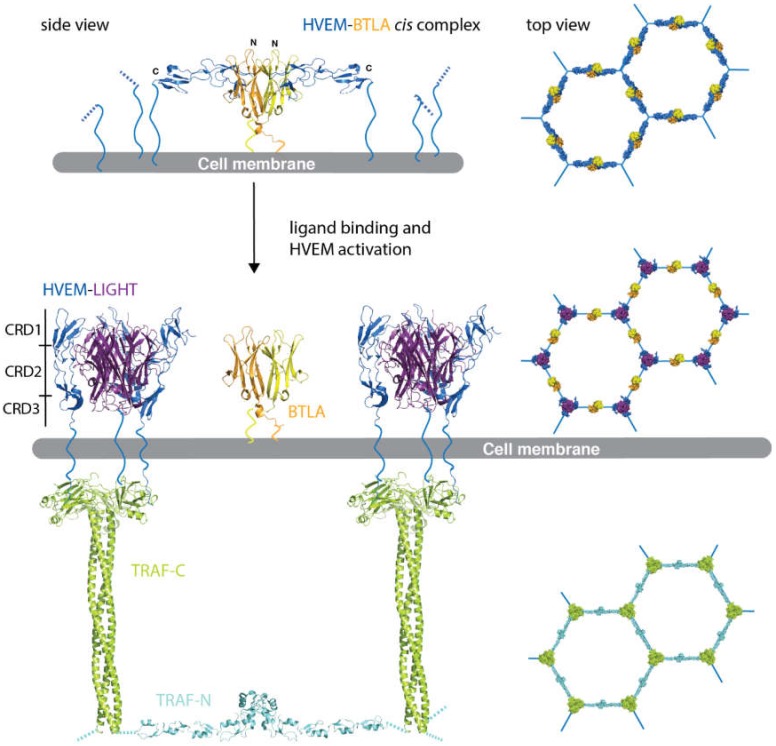
Regulation of herpes virus entry mediator (HVEM) signaling by B- and T lymphocyte attenuator (BTLA). The structure of the HVEM-BTLA *cis* complex (based on PDB ID: 2AW2) is shown in side view in cartoon representation (top left). The HVEM monomers are shown in blue and monomers of the BTLA dimer in yellow and orange respectively. BTLA aids the oligomerization of HVEM. The complexes may arrange in a hexagonal lattice shown on the right in surface representation (top view). Trimeric LIGHT binding to HVEM activates the receptors to form the LIGHT-HVEM complexes shown in cartoon representation (PDB ID: 4RSU). LIGHT is shown in magenta and HVEM in blue. The CRD1-3 are labelled in the left complex. The receptor ligand complexes may also cluster to form a hexagonal lattice shown on the right in top view. The HVEM-LIGHT complexes are bound to TNF related associated factor (TRAF) in the cytosol shown in chartreuse. TRAF trimerization is initiated by LIGHT binding and HVEM activation. This then leads to the dimerization of the TRAF-N domain shown in cyan. The TRAF complexes also form a hexagonal lattice shown on the right in surface representation. The TRAF complex was modeled from several structures as described earlier [14].

BTLA is an immune-globulin superfamily (IgSF) glycoprotein with two immunoreceptor tyrosine-based inhibitory motifs [46]. BTLA is an inhibitory receptor on T lymphocytes with similarities to cytotoxic T lymphocyte-associated antigen 4 (CTLA-4) and programmed death 1 (PD-1). The crystal structure of the HVEM-BTLA complex shows a heterotetramer consisting of a dimer of HVEM-BTLA heterodimers [47] (Figure 3) that resembles the antiparallel dimer of TNFR1 shown in Figure 1d. We believe that it is the heterotetramer seen in the crystal structure that represents the *cis* complex on the cell surface and not the model that was put forward in the original study [47]. CD160 and gD are similar to BTLA in that they also contain an Ig fold. BTLA and gD are monomeric in solution, but it has been shown that both may form dimers on the membrane, giving biological importance to the observed crystal structure [48,49]. On the basis of these data, we propose that the HVEM antiparallel complexes with BTLA may arrange in a hexagonal lattice on the cell surface representing the receptor quiescent state (Figure 3). Since BTLA is a type I transmembrane protein similar to HVEM, both can be co-expressed and anchored to the cell by their C-terminal ends. In the *cis* configuration BTLA does not interfere with LIGHT or LT-α binding, instead it serves to facilitate HVEM oligomerization on the cell surface and to inhibit the ligand independent activation of HVEM. In contrast to BTLA, the CD160-HVEM structure shows a 1:1 complex and it has a lower affinity for HVEM than BTLA [50]. There are conflicting data on whether CD160 is also monomeric on the cell surface [51,52,53]. If confirmed, a monomeric CD160 bound in *cis* may function to limit HVEM oligomerization on the cell surface in the above model. This shines potentially new light onto BTLA, CD160 function. Rather than being true ligands, they serve as regulatory proteins modulating HVEM oligomerization and control receptor activation. In addition to *cis* interactions, both BTLA and CD160 can also interact in *trans* adding to the complexity of HVEM regulation [49].

It is important to note here that the viral protein HCMV UL141 that blocks TRAIL-R2 surface expression interacts with TRAIL-R2 via its Ig-like domain. This highlights a potentially more extensive relationship between the Ig and TNF superfamilies then previously appreciated. As is shown in Table 1, there are many TNFRSF receptors with three or fewer CRD domains that could potentially utilize a co-regulatory protein to aid their oligomerization on the cell surface.

## 7. Conclusions

We have argued that the probability of random receptor arrangement on the cell surface matching up with randomly arranged membrane bound ligands is extremely low. Membrane bound ligands are more efficient at receptor activation because they allow the simultaneous activation of multiple receptors. However, for that to happen, the receptors and their corresponding ligands need to be arranged on the cell surface in a highly ordered manner with matching geometry. This should apply to not only members of the TNF receptor superfamily but to every clustered receptor activated by membrane bound ligands. We discussed how TRAIL-R1/R2 and other TNFRSF members cluster on the cell surface forming highly ordered hexagonal lattices. The available experimental data supports a receptor quiescent state of a hexagonal lattice made up of trimers of antiparallel dimers. For receptors like HVEM that are unable to dimerize on their own, co-regulatory proteins may aid receptor oligomerization on the cell surface and modulate receptor function. It remains to be seen if this more broadly applies to other TNFRSF receptors. The same process can also be exploited by viral proteins to modulate immune function. The antiparallel arrangement not only prevents accidental receptor activation but places the receptors with the right geometry for the downstream signaling partners to assemble. There may be exceptions but the same geometry seems to apply to both the death receptor family and for the TRAF-interacting TNFRSF members. Having the same scaffolding geometry can allow the switching between apoptosis and NFκB activation for the same receptor such as TNFR1 depending on the downstream binding partners.

Many questions still remain about the mechanism of TNFRSF signaling, but the presented model provides important insights into TNFRSF biology and for the design of better targeted therapies at the molecular level for cancer and other immune mediated diseases. Antibodies that lock in the antiparallel dimer provide an effective strategy for creating antagonist therapeutics against TNFRSF receptors. On the other hand, effective agonism may include both ligand and antibody-based approaches that utilize the hexagonal model of activation.

## Figures and Tables

**Table 1:** Tumor necrosis factor receptor superfamily (TNFRSF) members, their ligands, and intracellular binding partners.

TNFRSF Receptor (TNFRSF#, Other Names)	Number of CRD^‡^	Intracellular Binding Partner	TNFSF Ligand (TNFSF#, Other Names)
Death receptors			
TNFR1 (1a, CD120a)	4	TRADD, FADD, RIP	TNF (2, TNF-α), LTα (1, TNF-β), LTβ (3)
Fas (6, CD95)	3	FADD	FasL (6, CD178)
TRAILR1 (10A, DR4, CD261)	3^¶^	FADD, TRADD, RIP	TRAIL/Apo2L (10, CD253)
TRAILR2 (10B, DR5, CD262)	3^¶^	FADD, TRADD, RIP	TRAIL/Apo2L (10, CD253)
NGFR (16, p75NTR, CD271)	4	NADE	NGF (not a TNFSF member)
DR3 (25 or 12, TRAMP)	4^¶^	TRADD, FADD	TL1A (15, VEGI), TWEAK (12)
DR6 (21, CD358)	4	TRADD, RIP	N-APP (not a TNFSF member)
EDAR	3^¶^	EDARADD	EDA-A1
Receptors with TRAF-interacting motif
TNFR2 (1b, CD120b)	4	TRAF1-3	TNF (2, TNF-α), LTα (1, TNF-β)
LTβR (3)	4	TRAF2-4, TRAF5	LTα (1, TNF-β), LTβ (3) as LTαβ_2_, LTα_2_β
OX40 (4, CD134)	4^¶^	TRAF1-3, TRAF5, TRAF6	OX40L (4, CD252)
CD40 (5)	4	TRAF1-3, TRAF5, TRAF6	CD40L (5, CD154)
CD27 (7)	3	TRAF2, TRAF3, TRAF5	CD27L (7, CD70)
CD30 (8)	6	TRAF1-3, TRAF5	CD30L (8, CD153)
4-1BB (9, CD137)	4	TRAF1-3	4-1BBL (9, CD137L)
RANK (11A, CD265)	4	TRAF1-3, TRAF5, TRAF6	RANKL (11, TRANCE, CD254)
Fn14 (12A, TWEAKR; CD266)	1	TRAF2, TRAF6	TWEAK (12)
TACI (13B, CD267)	2	TRAF2-3, TRAF5, TRAF6	APRIL (13, CD256)
BAFFR (13C, BR3, CD268)	1	TRAF2, TRAF3, TRAF6	BAFF (13B/20, BLys, THANK, CD257)
HVEM (14, CD270)	3	TRAF1-3, TRAF5	LIGHT (14, CD258), LT-α (1, TNF-β)
BCMA (17, CD269)	1	TRAF1-3, TRAF5, TRAF6	APRIL (13, CD256), BAFF (13B/20, BLys, THANK, CD257)
GITR (18, AITR, CD357)	3	TRAF1-3	GITRL (18, AITRL, TL6)
TROY (19, TAJ)	3^¶^	TRAF1-3, TRAF5	?
RELT (19L)	1	TRAF1	Not known
XEDAR (27)	3^¶^	TRAF1, TRAF3, TRAF6	EDA-A2
Decoy receptors		n/a	
TRAILR3 (10C, DcR1, CD263)	3^¶^		TRAIL/Apo2L (10, CD253)
TRAILR4 (10D, DcR2, CD264)	3^¶^		TRAIL/Apo2L (10, CD253)
OPG (11B)	4		TRAIL/Apo2L (10, CD253), RANKL (11, TRANCE, CD254)
DcR3 (6B)	4		FasL (6), TL1A (15, VEGI), LIGHT (14, CD258)

‡: Uniprot (uniport.org) assignments were used to determine the number of cysteine-rich domains (CRD); ^¶^: Contains truncated CRD domains.
